# A Sensor-Based System for Fault Detection and Prediction for EV Multi-Level Converters

**DOI:** 10.3390/s23094205

**Published:** 2023-04-22

**Authors:** Răzvan Gabriel Prejbeanu

**Affiliations:** 1 Department of Automatic Control and Electronics, University of Craiova, 200585 Craiova, Romania; razvan.prejbeanu@edu.ucv.ro; 2 INDAELTRAC Ltd., 200385 Craiova, Romania

**Keywords:** monitoring systems, fault diagnosis, multilevel inverter, multi carrier PWM, total harmonic distortion, induction motor

## Abstract

Power electronic converters and alternating current motors are the actual driving solution applied to electric vehicles (EVs). Multilevel inverters with high performance are modern and the basis for powering and driving EVs. Fault component detection in multilevel power converters requires the use of a smart sensor-based strategy and an optimal fault analysis and prediction method. An innovative method for the detection and prediction of defects in multilevel inverters for EVs is proposed in this article. This method is based on an algorithm able to determine in a fast and efficient way the faults in a multilevel inverter in different possible topologies. Moreover, the fault detection is achieved not only for a single component, but even for several components, if these faults occur simultaneously. The detection mechanism is based on the analysis of the output current and voltage from the inverter, with the possibility of distinguishing between single and multiple faults of the power electronic components. High-performance simulation programs are used to define and verify the method model. Additionally, with this model, harmonic analysis can be performed to check the correctness of the system’s operation, and different fault scenarios can be simulated. Thus, significant results were obtained by simulation on various topologies of multilevel converters. Further, a test bench was developed in order to verify some failure situations on a three-level inverter.

## 1. Introduction

Electric vehicles have become a reality in the conditions of the exhaustion of fossil fuel resources. One field where power electronics is widely used today is the EV industry through the well-known binomial: static power converter-electric motor. The use of systems comprising static converters with electronic power components and alternating current motors, asynchronous or synchronous, represents the modern solution in the case of EVs [1,2,3]. Intelligent sensor systems optimize standard (classical) measuring structures and are used to acquire different electrical parameters. This option may be cheaper and easier to implement on a vehicle, given the cost and space limitations. Multilevel inverters have major advantages over traditional two-level inverters, such as compact converter size, low harmonic distortion (THD) due to the stepped output wave, and modular structure. One of the important problems of vehicle drive systems is to increase the efficiency of electric propulsion from the vehicle battery and increase the operating distance between two successive charges.

However, the reliability is the main problem in the field of systems driven by electronic power converters and electric motors, according to the conclusion of 93% of the respondents of a 2011 study in the field where electronic power converters are used [4]. However, the failure of electronic components also strongly affects the reliability of the entire system. The most frequent failures of the power components of the inverter are short circuit—continuity or circuit interruption—open circuit failures [5,6,7,8]. In most cases, short-circuit faults cause overcurrent conditions in the converters, which can be detected with standard sensor systems mounted on the output of the inverters. The occurrence of a short circuit fault disconnects the power source, grid, or battery to protect the associated components against damage. In contrast, the interruption of the power component generally does not cause the system to shut down; instead, it degrades performance, even leading to serious secondary failures in other parts of the system. Because the diagnosis of faults in inverters whose power components are interrupted is critical for the inverter [9] that can be used in the electric drive of vehicles, the issue of diagnosing faults in complicated systems is very important and actual [10,11,12,13].

Multiple diagnostic methods have been proposed for open-circuit failures of power components in inverters. The methods can be direct, by measuring electrical parameters, or indirect, by calculating some parameters. Analysis methods can be classified into methods based on voltage measurement and methods based on current measurement. Hall or flux gate transducers are used to measure these electrical parameters.

A quantitative, indirect way to determine the open-circuit fault uses the Park transformation [14]. Fault determination methods based on the Park transform are usually current-based methods. The description of the use of this method that proposes the Park vector technique is presented in [15,16,17]; the principle of this technique is based on following the trajectory of the currents of the Park system (*i_d_*, *i_q_*). When the system is working correctly, the trajectory has the shape of a circle, and in the case of an open-circuit failure of the insulated gate bipolar transistors (IGBTs), the circle can narrow to a semicircle. The position of this trajectory within the (*d*–*q*) axis system makes it possible to calculate the angle ranges of the space vectors to locate the faulty IGBT.

Another technique based on the calculation of the average current value (*id_average_*, *iq_average_*) is used to accurately calculate the control angle and to identify the interrupted IGBT, open-circuit fault. The technique based on the spectral analysis of the stator currents is a common method [18,19,20]. This technique is based on the harmonic analysis of each phase current. The amplitude and rank of each harmonic can be used in fault detection and localization. The analysis of the first harmonic shows that the difference between the correct operating state and the open-circuit failure state is found in the zero-order harmonics that represent the presence of the DC component in the signal.

A method based on voltage measurement is presented in [21]. The fault diagnosis method is based on sampling the zero-voltage vector for current sampling and reconstructing the three-phase current. In [22], a multiscale adaptive fault diagnosis method is presented. The method is based on the preprocessing of the symmetric signal reconstruction and has been recommended to diagnose microgrid inverter faults under variable load conditions. In [23], a strategy was developed for the identification of open-circuit faults by constructing standardized variables for the failure modes. In [24], a fault detection model based on the determination of the line voltage envelope from the inverter output is presented. Other methods are based on the measurement of the instantaneous voltage error in the open-circuit fault situation [25]. However, methods based on voltage measurement need additional voltage sensors and a complex central analysis unit. These systems are sensitive to load changes [26].

Open-circuit faults are more difficult to detect and cannot be detected by the standard protection system. This type of failure induces constraints in the operation of the EV if the driver is not notified and can eventually result in a stop that can be even dangerous, leading to accidents. Such a defect can cause secondary failures in other vehicle components that can lead to high repair costs [27].

A method based on hybrid logic for the dynamic model of the converter was used to estimate open-circuit faults by analyzing the current of the power network [28]. Other solutions for diagnosing open-circuit faults were based on rotor current variation [29]. Monitoring the variation of the current in the neutral circuit of the DC link of a T-type multilevel converter was proposed as a method in [30].

Another solution, proposed in [31], is based on the estimation of current thresholds, measured with Hall transducers applicable for inverters considered as a three-phase voltage source. The open-circuit fault diagnosis algorithms are based on the use of adaptive thresholds. Park’s transformation method is necessary to compare the calculated and measured values with the set threshold to perform the fault diagnosis. It is difficult to guarantee the accuracy of these fault diagnosis methods when applied to real systems. When the load changes, it is difficult to set the threshold value to a constant, because the amplitude of the load current cannot be anticipated.

Uncertainty is a big challenge in predictive failure diagnosis of inverters. The uncertainty can be caused by several factors, such as the width of the frequency band or the noise of the signal provided by the sensors, or the method used and an incomplete mathematical model. The diagnostic methods in [32,33,34,35,36,37] are focused on the open-circuit fault of a two-level inverter. However, the three-level inverter provides more power for the same power components than a two-level inverter, resulting in a more complex topology and lower reliability. Therefore, the possibility of faults in a three-level inverter is greater, and the types of faults are more diverse. In [38,39,40], the feature needed to detect multi-level inverter faults was extracted by fast Fourier transform (FFT) and determined by support vector machine (SVM). In these works, the fault characteristics were extracted from the output voltage waveform but only for purely resistive loads. In addition, the FFT method is used for signal analysis [41]. This analysis does not perform well in the transient regime of the system and cannot accurately determine the moment of fault occurrence.

Only a few studies deal with the determination of faults in the intermediate DC circuits [42,43] of inverters. These types of defects become significant and relevant in the case of using multilevel inverters for driving EVs due to the large number of capacitors.

To solve these uncertainty problems, an innovative fault diagnosis methodology is proposed in this work. This method is based on the use of existing systems of voltage and current sensors in conventional three-phase multilevel inverters, without the use of additional ones. The measured electrical signals are used both in the direct current charging circuit and the control and regulation system of the multilevel inverters [44,45]. The proposed fault diagnosis method achieves the detection of all types of faults for all power components, IGBTs, diodes or capacitors, in multilevel inverters. The system is useful both for diagnosing open-circuit faults and short-circuit faults as well as for faults in the direct current intermediate circuit.

To the best of our knowledge, the method proposed in this paper is original and has not yet been implemented practically anywhere in this way. The method is the same and valid for all types of components—active or passive. The practical way of applying the method is carried out in four sections: receiving and analyzing the signals from the transducers (sensors), identifying the types of faults, extracting the characteristics of the faults, and respectively transmitting and making decisions at the level of the control system.

The use of techniques based on artificial intelligence is significant for the future completion of the method of detecting faults in multilevel inverters. Currently, there is no comprehensive overview of the application of techniques based on artificial intelligence in the detection and diagnosis of faults specific to the electric drive of EVs. However, determining faults in multilevel inverter and asynchronous motor drive systems for electric vehicles could be implemented using several common architectures, including autoencoder (AE), recurrent neural network (RNN), generative adversarial network (GAN) or deep trusted network (DBN).

The rest of this paper is organized as follows. Section 2 deals with technical considerations. Section 3 presents the principle and implementation of the proposed method of fault detection, and in Section 4 the results of simulations are presented, and some discussions are provided. Section 5 is devoted to the experimental validation. Finally, in Section 6, concluding remarks are presented.

## 2. Multi-Level Inverters—Technical Considerations

The automotive industry has now started to demand and use more converters with rated power up to several tens and even hundreds of kW with good efficiency, low EMI, and with as minimal a distortion output waveform as possible.

The special structure of multi-level voltage inverters allows high voltage and high currents to be obtained at the output. The structure and control system of the multilevel inverters significantly reduce the amplitude of the upper voltage harmonics and achieve an optimal output voltage and power transfer without the use of an adapter or isolation transformer.

**Figure 1:**
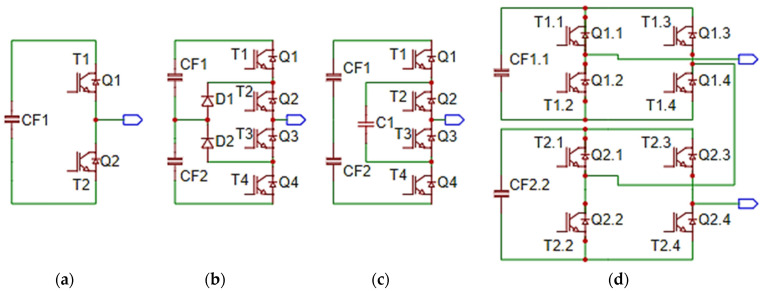
Topologies of multilevel inverters: (**a**) two-level inverter; (**b**) three-level neutral-point clamped (NPC); (**c**) flying-capacitor; (**d**) cascaded H-bridge.

The standard topologies for inverters are shown in Figure 1. Figure 1a shows the structure of a two-level inverter. The three-level diode neutral point clamped inverter needs only one direct current circuit, and the voltage levels are obtained by using several capacitors connected in series (Figure 1b). Flying capacitor inverter topology is very complicated and practically only three-level diode inverters are available on the market. The flying capacitor multilevel inverter requires isolated DC sources for each DC bus (Figure 1c). This aspect determines a very complicated, large and expensive direct current bus structure. The cascaded multilevel inverter requires DC sources isolated for each intermediate DC circuit (Figure 1d). These considerations make the DC-link structure very complicated.

Three-level inverters in NPC topology are the most used types of multilevel inverters. Moreover, power module manufacturers have started manufacturing and launching on the market such structures for multilevel inverters made in this topology, one of the main reasons being the reduction of the number of power modules. Thus, in order to implement and to test the proposed method, the present study was conducted on the best represented structure/topology. Another reason is the combination of qualities that the output voltage wave has in comparison with other topologies. With such an inverter, the output voltage has values from *V_dc_/2*, 0 and −*V_dc_/2* with respect to the neutral point.

**Figure 2:**
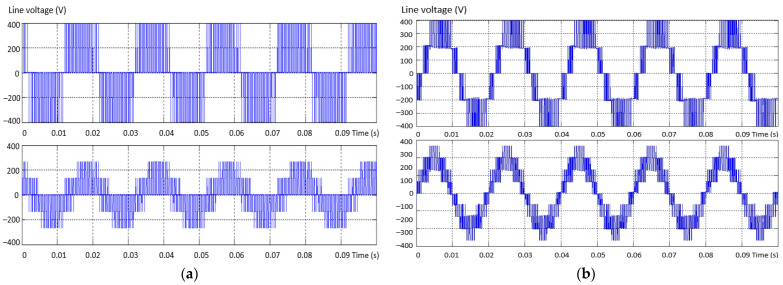
The shape of the phase and line voltages for: (**a**) 2L and (**b**) 3L inverter.

The modern solution for obtaining a voltage waveform with a low content of harmonics is the use of multilevel inverters instead of the usual ones with only two levels. The voltage waveform at the output of the two-level inverter is produced by using the the pulse width modulation (PWM) method. In this situation, there is an increase in the line and phase voltage distortion values at the output of the inverter, and this determines a high THD value of the voltage (Figure 2a). In the three-level inverter, the output voltage and current are much closer to sinusoidal, less distorted, and the THD is better (Figure 2b).

The efficiency at full load is better in the three-level inverter than in the two-level inverter. The high efficiency of the inverter determines a better use of the energy from the vehicle’s battery. Better efficiency at rated power also means a smaller heat sink and better reliability. The efficiency of the three-level inverter at low power is also improved. The *P*/*P_max_* value is reduced by 50%. A multilevel inverter has more components than a 2L inverter, and at first sight, it seems that the reliability of the system should be lower. Considering the use of an increasingly higher voltage in EV batteries the IGBTs of the 2L inverter must be of higher voltage with higher losses but also with related elements of large dimensions (heating, ventilation, heatsinks, etc.). By using some power FET transistors but low voltage in a multilevel inverter structure, the switching frequency can be increased above the 20 kHz limit value for high-voltage IGBTs with an increase in the inverter performance. The reliability of the components drops drastically if they are used at the limits of the electrical and thermal parameters. In these conditions, the difference between the material costs of the two classes of inverters tends to narrow. From a specific constructive point of view and from an economic point of view, a 3L inverter arm made with 600 V silicon-based IGBTs is 25% cheaper than a 2L inverter arm with 1200 V silicon IGBTs.

**Figure 3:**
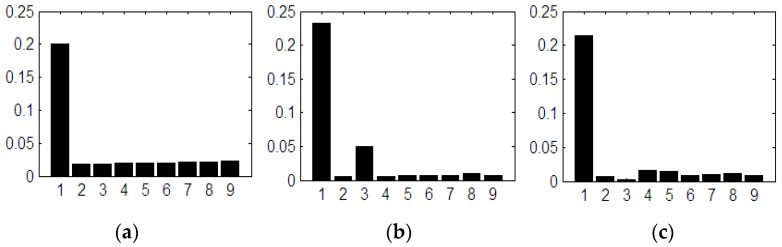
The harmonic spectra corresponding to: (**a**) the sinusoidal modulation; (**b**) vector modulation and (**c**) optimally modulated signal (acquired).

If the number of PWM output pulses is smaller, the amplitude of the fundamental is reduced, and in this case the number and effect of the lower harmonics is more pronounced. If the number of pulses is high, the influence of higher order harmonics becomes more important. These effects are presented by measuring the stator currents of the vehicle’s electric motor. The higher order current harmonics are filtered by the inductance of the asynchronous electric motor, and this phenomenon is represented in Figure 3 by measuring the phase current and calculating the THD for the 2L inverters.

The effects of harmonics on the asynchronous motor are unfavorable and produce limitations or reductions in the operating capacity of the EV. These effects are thermal effects or mechanical effects produced by harmonic torques that can be stationary or pulsating. An important effect of the output harmonics of the inverters on the asynchronous motor is the increase in its heating due to the additional losses, mainly the copper losses associated with the harmonic currents. Global losses can increase due to the presence of harmonics and can consequently reduce the active torque, due to parasitic torques and increased temperature. A solution to this problem could be the filtering of these harmonics by introducing a sine filter at the output of the inverter [46,47]. This solution cannot be effectively applied in automotive solutions due to significant additional losses from the sine filter at the output, which would reduce the distance traveled by EVs with batteries.

The proposed multilevel inverter fault analysis method applies to any multilevel inverter structure and topology, as the calculation relationships are specific to both the topology and the number of levels. The physical model of the components, calculations and load parameters have no influence on the proposed method.

## 3. Principle and Implementation of the Fault Detection Method

### 3.1. Method Principle

The power converter is subjected to various internal and external constraints that can result in its failure during operation. Because of their sensitivity, IGBT semiconductors are the most exposed to failure. For electronic components, the following two types of important faults can be registered, as follows:

(a) Short circuit. Short-circuit faults affecting both IGBTs and power diodes in the circuit are the most serious faults. In the presence of such a fault, the current reaches limits that can cause destruction by short-circuiting and melting the chip. If the detection of this type of failure is not performed quickly (less than 10 μs) and no specific measures are taken, then the IGBT switch that is still active on the same inverter arm suffers the same destruction phenomenon, and thus the entire inverter arm is short-circuited.

(b) Open-circuit fault, interrupted. Open-circuit faults affecting IGBT power switches can occur when, for whatever reason, the IGBT is disconnected, damaged, or has had a problem with the base control signal. This type of failure is very difficult to detect directly because the motor can continue to work, but with a performance degradation due to the occurrence of fluctuations in mechanical parameters (speed and torque), as well as an imbalance of currents. In this situation, the currents of the other two valid arms take high values because the system tries to maintain the average torque and speed. Starting in the presence of this type of fault may not always be possible.

**Figure 4:**
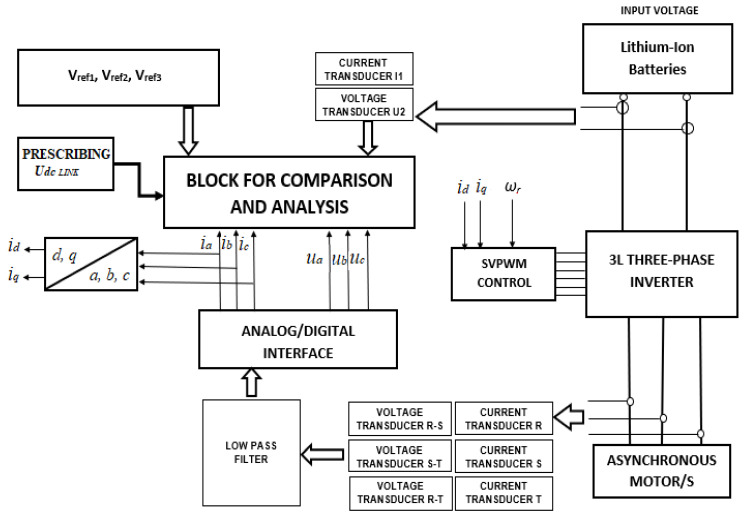
Block diagram of the fault reporting and treating system.

A block diagram of the fault detection and treatment system is shown in Figure 4. Its operation is based on the acquisition and comparison of the electrical parameters (currents and voltages) associated with a 3L three-phase inverter, with the values obtained from the calculation or directly prescribed and representing the reference values. The values of the phase currents and line voltages provided by the inverter are used to control the asynchronous motor with the use of known transformations (Clarke and Park) applied to the best performing direct torque control (DTC) techniques, field oriented control (FOC). At the same time, these values can be used to determine the faulty power components in the inverter. To reduce the risk of false alarm signals, measures were taken both at the level of electrical signals (filters) and at the level of signal acquisition methods. In Figure 4, a block that contains low-pass filters and that removes the spikes produced by switching the power converter on the EV was introduced. To obtain signals with the highest possible accuracy and without errors, an optimized analog-to-digital conversion method was used. The time intervals used in the matrices of measured and predictive values are large enough to allow multiple readings. In each interval, readings are made, and those values that are lower or higher than the average of the values read in the measurement interval are removed according to a predetermined error.

The basic principle of space vector PWM (SVPWM) depends on synthesizing the reference voltage vector by the time average of the two vectors produced by the inverter. The reference voltage vector is the required control voltage, which should be supplied as required by the application. The SVPWM technique is based on the approximation of the rotating space vector of the reference voltage. The vector amplitude and phase angle can be determined from the instantaneous values of the voltages. If the quantities are sinusoidal and balanced, the vector will rotate by a fixed angle and have a constant amplitude.

For the three-phase system of an inverter, there are 27 switching states. Each of these switching states can be represented as a vector:

(1) $$\vec{v}=V_{\alpha }+jV_{\beta }=\frac{2}{3}\;(V_{a}e^{j0}+V_{b}e^{\frac{j2\pi }{3}}+V_{c}e^{\frac{j4\pi }{3}})$$

with the angle:

(2) $$\theta =\tan^{-1}\left(\frac{V_{\alpha }}{V_{\beta }}\right)$$

The voltages *V_a_*, *V_b_*, and *V_c_* are the reference three-phase voltages measured with voltage transducers, and *V_α_* and *V_β_* are the components of the reference vector in the fixed coordinate system (*α*, *β*).

By using the Clarke transformation, the three-phase *a*-*b*-*c* coordinate system is transformed into an orthogonal stationary reference system (*α*, *β*), which is useful in identifying the sector by the following expression:

(3) $$\left[\begin{array}{c} V_{\alpha }\\ V_{\beta } \end{array}\right]=\left[\begin{array}{ccc} 1 & -\frac{1}{2} & -\frac{1}{2}\\ 0 & \frac{\sqrt{3}}{2} & -\frac{\sqrt{3}}{2} \end{array}\right]\left[\begin{array}{c} V_{a}\\ V_{b}\\ V_{c} \end{array}\right]$$

Further using the Park transform, the transition from the fixed two-axis orthogonal system (*α*, *β*) to the two-dimensional mobile orthogonal system (*d*, *q*) is achieved.

(4) $$\left[\begin{array}{c} V_{d}\\ V_{q} \end{array}\right]=\left[\begin{array}{cc} \cos\left(\theta \right) & \sin\left(\theta \right)\\ -\sin\left(\theta \right) & \cos\left(\theta \right) \end{array}\right]\left[\begin{array}{c} V_{\alpha }\\ V_{\beta } \end{array}\right]$$

or

(5) $$\left\{\begin{array}{c} V_{d}=V_{\alpha }\;cos\theta +V_{\beta }\;sin\theta \\ V_{q}=V_{\beta }\;cos\theta -V_{\alpha }\;sin\theta \end{array}\right.$$

In a similar manner, the three-phase currents are acquired by the system, and the components of the two-dimensional stationary orthogonal system are obtained by using the Clarke and Park transformations:

(6) $$\left[\begin{array}{c} i_{\alpha }\\ i_{\beta } \end{array}\right]=\left[\begin{array}{ccc} 1 & -\frac{1}{2} & -\frac{1}{2}\\ 0 & \frac{\sqrt{3}}{2} & -\frac{\sqrt{3}}{2} \end{array}\right]\left[\begin{array}{c} i_{a}\\ i_{b}\\ i_{c} \end{array}\right]$$

respectively:

(7) $$\left[\begin{array}{c} i_{d}\\ i_{q} \end{array}\right]=\left[\begin{array}{cc} \cos\left(\theta \right) & \sin\left(\theta \right)\\ -\sin\left(\theta \right) & \cos\left(\theta \right) \end{array}\right]\left[\begin{array}{c} i_{\alpha }\\ i_{\beta } \end{array}\right]$$

or

(8) $$\left\{\begin{array}{c} i_{d}=I_{\alpha }\;cos\theta +I_{\beta }\;sin\theta \\ i_{q}=I_{\beta }\;cos\theta -I_{\alpha }\;sin\theta \end{array}\right.$$

In these relations, *i_d_*, *i_q_* are parameters of the rotating reference system (spinner), *I_α_*, *I_β_* are the orthogonal stationary reference frame parameters, and *θ* is the rotating angle.

The obtained *i_d_* and *i_q_* parameters are further used in the SVPWM control block and, together with the motor speed signal/s, are used to obtain the required control pulses for the power inverter IGBTs. After performing the application-specific calculations (control of the asynchronous motor used to drive an EV) and with the aim of performing the fault analysis, it is necessary to “restore” the three-phase voltage and output current values used as a reference for the fault prediction system.

In this case, the computations are continued to obtain the reference values *u_a_*, *u_b_*, *u_c_* by using the inverse Park^−1^ and Clarke^−1^ transformations. The inverse Park^−1^ transformation is applied to the input values with the position given by the angle *θ*:

(9) $$\left[\begin{array}{c} u_{\alpha }\\ u_{\beta } \end{array}\right]=\left[\begin{array}{cc} \cos\left(\theta \right) & -\sin\left(\theta \right)\\ \sin\left(\theta \right) & \cos\left(\theta \right) \end{array}\right]\left[\begin{array}{c} u_{d}\\ u_{q} \end{array}\right]$$

where *u_α_*, *u_β_* are stationary orthogonal reference frame parameters.

The transformation of the stationary system of axes (*α*, *β*) into the stationary three-phase system with reference to the stator is presented by the relation:

(10) $$\left\{\begin{array}{c} u_{a}=u_{\alpha }\\ u_{b}=\frac{-u_{\alpha }+\sqrt{3}u_{\beta }}{2}\\ u_{c}=\frac{-u_{\alpha }-\sqrt{3}u_{\beta }}{2} \end{array}\right.$$

or

(11) $$\left[\begin{array}{c} u_{a}\\ u_{b}\\ u_{c} \end{array}\right]=\left[\begin{array}{cc} 1 & 0\\ -\frac{1}{2} & \frac{\sqrt{3}}{2}\\ -\frac{1}{2} & -\frac{\sqrt{3}}{2} \end{array}\right]\left[\begin{array}{c} u_{\alpha }\\ u_{\beta } \end{array}\right]$$

The calculation relations for the three-phase output voltages and currents are:

(12) $$\begin{array}{ll} v_{a}\left(t\right)=V_{m}\sin\left(\omega t+\theta \right) & i_{a}\left(t\right)=I_{ma}\sin\left(\omega t+\theta \right)\\ v_{b}\left(t\right)=V_{m}\sin\left(\omega t-\frac{2\pi }{3}\right) & i_{b}\left(t\right)=I_{mb}\sin\left(\omega t-\frac{2\pi }{3}+\theta \right)\\ v_{c}\left(t\right)=V_{m}\sin\left(\omega t+\frac{2\pi }{3}\right) & i_{c}\left(t\right)=I_{mc}\sin\left(\omega t+\frac{2\pi }{3}+\theta \right) \end{array}$$

The duty cycles in the three-phase system are obtained as:

(13) $$d_{a}\left(t\right)=\left|m\sin\left(\omega t\right)\right|,\;d_{b}\left(t\right)=\left|m\sin\left(\omega t-\frac{2\pi }{3}\right)\right|,\;\;d_{c}\left(t\right)=\left|m\sin\left(\omega t+\frac{2\pi }{3}\right)\right|$$

where *m* is the modulation factor and represents the ratio between the PWM frequency and the fundamental of the output frequency, and *m_f_* = *fs*/*fi*, for which *m_f_* should be an odd integer.

If *m_f_* is not an integer, then subharmonics are present in the output voltage. If *m_f_* is not odd, there is a DC component or even harmonics in the output voltage. For normal steady-state operation, 0 < *m* ≤ 1.

The value of the neutral current is:

(14) $$i_{0}\left(t\right)=d_{a}\left(t\right)\times i_{a}\left(t\right)+d_{b}\left(t\right)\times i_{b}\left(t\right)+d_{c}\left(t\right)\times i_{c}\left(t\right)=\;=\left|m\sin\left(\omega t\right)\right|\times I_{ma}\sin\left(\omega t+\theta \right)+\left|m\sin\left(\omega t-\frac{2\pi }{3}\right)\right|\times I_{mb}\sin\left(\omega t-\frac{2\pi }{3}+\theta \right)+\;+\left|m\sin\left(\omega t+\frac{2\pi }{3}\right)\right|\times I_{mc}\sin\left(\omega t+\frac{2\pi }{3}+\theta \right)$$

In a three-phase alternating system, the voltage measured from phase to ground can be written as the addition of the voltage from phase to load neutral and from load neutral to system ground. By definition, the common mode voltage is the voltage from the neutral point of the load to the system ground. Since in a balanced system the sum of all three phase-to-neutral voltages is zero, the neutral-to-ground voltage (common-mode voltage) can be defined as the phase-to-ground voltage.

For inverters, *m_f_* must be 3 or a multiple of 3.

(15) $$V_{peack}=m\frac{V_{dc}}{2}$$

where *V_peack_* is the peak value of the fundamental component of the phase-to-neutral voltage, and *V_dc_* is the d.c. voltage in the DC link. For three-phase space vector SVPWM,

(16) $$V_{peack}=m\frac{V_{dc}}{\sqrt{3}}$$

The scheme of the 3-L NPC inverter was adopted for the application of the method. This topology is the most used nowadays. Both the faults of the power components, the IGBTs, and the faults appearing in the intermediate circuit are analyzed. This type of comprehensive strategy is less addressed in the specialized documentation and specifications of inverters. The analysis is carried out both at short circuit and at the interruption of these components.

**Figure 5:**
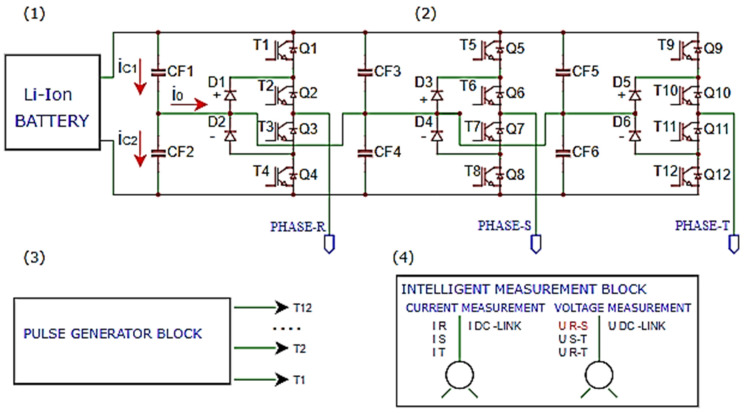
Schematic of the main circuit of the three-level NPC inverter.

The main circuit of the three-level NPC inverter is shown in Figure 5 and consists of:

(1) Li-Ion battery pack
(2) the power circuit of the three-phase inverter 3L
(3) the control pulse generator block
(4) the block of current and voltage Hall transducers

The failure of some capacitors causes an imbalance that appears in the intermediate circuit and causes the appearance of a current *i*_0_ through the neutral point of the scheme. The value of *ic*_1_ is the current of the upper capacitor *C*_1_, and *ic*_2_ is the current of the lower capacitor *C*_2_.

Considering that the values of the capacitors are equal, *C*_1_ = *C*_2_ = *C_Fk_*. The values of the currents *ic*_1_, *ic*_2_ and *i*_0_ are obtained as shown below:

(17) $$I_{C}=C\frac{dU}{dt}$$

and respectively

(18) $${i_{C}}_{1}\;=\;C\frac{d\left(\frac{V_{dc}}{2}-v_{0}\right)}{dt},\;{i_{C}}_{2}\;=\;C\frac{d\left(\frac{V_{dc}}{2}+v_{0}\right)}{dt},\;i_{0}\;=\;i_{C1}\;-\;i_{C2}=-2C\frac{d\left(v_{0}\right)}{dt}$$

The variation in the voltage at the neutral point depends on the value of the current at the neutral point. The relationship between neutral point current and neutral point voltage is described by:

(19) $$u_{C}=\frac{1}{C}\int idt$$

(20) $$v\left(t\right)=-\frac{1}{2C}\int_{0}^{t}i_{0}\left(\tau \right)d\tau +V_{0}\left(0\right)$$

where *V*_0_(0) is the initial value.

From the relations above, it can be seen that obtaining the neutral point voltage can be transformed into a problem of the current in the neutral point. In this way, the expression for the neutral point current under any condition is deduced.

In two-level converters, +*V_dc_* and −*V_dc_* voltage pulses are used for each half-cycle, called bipolar switching, and in three-level inverters, either +*V_dc_* or −*V_dc_* sequence is used for each half-cycle, called unipolar switching. Typically, a synthesized selective harmonic elimination–voltage source converter (SHE VSC) output waveform is constructed to have quarter-wave symmetry (QWS). One of the single-phase waveforms is defined by Fourier series decomposition and described by relation (21):

(21) $$V(\omega t)\;=\;a_{0}+\sum_{k=1}^{\infty }a_{k}\cos(2\pi f_{0}kt)+b_{k}\sin(2\pi f_{0}kt)$$

with *f*_0_ the frequency of the fundamental component, *k* the harmonic order, and *ω* = 2π*f*_0_.

The equation can then be rewritten as:

(22) $$V(\omega t)\;=\;a_{0}+\sum_{k=1}^{\infty }a_{k}\cos(\omega kt)+b_{k}\sin(\omega kt)$$

The coefficients of the above Fourier series *a*_0_, *a_k_*, *b_k_* can be determined from the following equations:

(23) $$a_{0}=\frac{1}{2\pi }\int_{0}^{2\pi }V_{dc}d\omega t$$

(24) $$a_{n}=\frac{1}{\pi }\int_{0}^{2\pi }V_{dc}\cos(k\omega t)\;d\omega t$$

(25) $$b_{n}=\frac{1}{\pi }\int_{0}^{2\pi }V_{dc}\sin(k\omega t)\;d\omega t$$

Under these conditions, it can be written:

(26) $$V(\omega t)\;=\;a_{0}+\sum_{k=1}^{\infty }c_{k}\sin\left(\omega kt+\varphi_{k}\right)$$

where *a_k_* = *c_k_* sin*φ_n_*; *b_k_* = *c_k_* cos*φ_n_*; *c_k_* = $\sqrt{a_{k}{}^{2}+b_{k}{}^{2}}$; *φ_k_* = $\tan^{-1}(\frac{a_{k}}{b_{k}}$) for *b_k_* > 0; *φ_k_* = $\tan^{-1}(\frac{a_{k}}{b_{k}}$) + 180° for *b_k_* < 0.

### 3.2. Method Implementation

The proposed implementation is carried out on the one hand by direct reading through the sensor system and on the other hand by calculating the predicted parameters. Thus, both the matrix of the real (measured) values and the matrix of the calculated values will be completed. It is important to synchronize the on-time reading system with the tracking of the completion timing of the matrix of calculated values.

The voltage level matrix is defined as:

(27) $$V_{i,j}^{u}\;=\;(v_{ij})$$

where

*i* = $\bar{1\ldots n}$—the line—represents the voltage values at the *λ_k_* level at “*p*” time interval,
*j* = $\bar{1\ldots p}$—column—represents the voltage values at the same moment in time
  (28) $$V_{i,j}^{u}\;=\;\left[\begin{array}{ccc} v_{11} & \cdots & v_{1p}\\ \vdots & \ddots & \vdots \\ v_{n1} & \cdots & v_{np} \end{array}\right]$$

All of the voltage levels of the *λ_k_* lines are equal in value and refer to the value of the inverter supply voltage.

(29) $$\sum_{k=1}^{n}\lambda_{\kappa }=2V_{DC}$$

(30) $$\sum_{k=1}^{p}t_{\kappa }=T_{\mathrm{sample reading cycle}}$$

**Figure 6:**
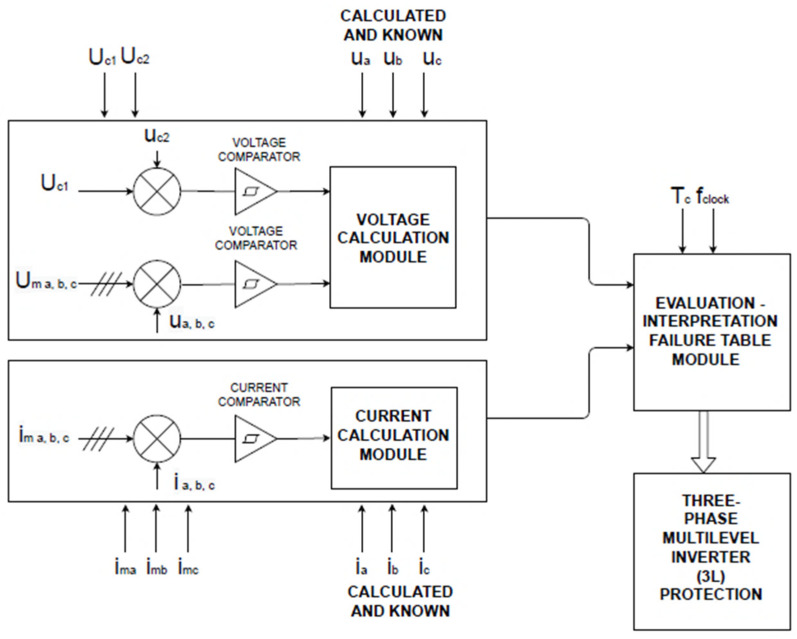
Detailed diagram of the “Analysis and comparison block”.

The comparison and analysis block are presented in Figure 6, as the main part of the block diagram has already been shown in Figure 4. The block is made both of hardware comparison modules for the quick determination of external faults and of software modules for calculating the predicted values and for determining the harmonic composition of the acquired signals.

The steps of the proposed working method can be summarized as follows:

- measure the output voltage and current at *t_k_* and fill in the value in the matrix, the table of measured values. The matrix of the measured values of voltages and currents is obtained;
- the voltage and current values are calculated according to the method of Park and Clarke transforms under the same time conditions *t_k_*. The matrix of calculated values of voltages and currents is then obtained;
- the comparative spectral analysis determines in real time the appearance of an open-circuit defect or an internal short circuit. The determined values are compared with the expected and known values, and in the off-time mode it is precisely determined which components are defective;
- each value obtained by calculation is compared with the measured one.

The parameters written in each position of the two matrices are then compared, position by position, taking into account an imposed deviation. The matrix of obtained deviations is analyzed according to the patterns of the diagrams depending on the accepted statistical differences deciding the type of occurred fault.

**Figure 7:**
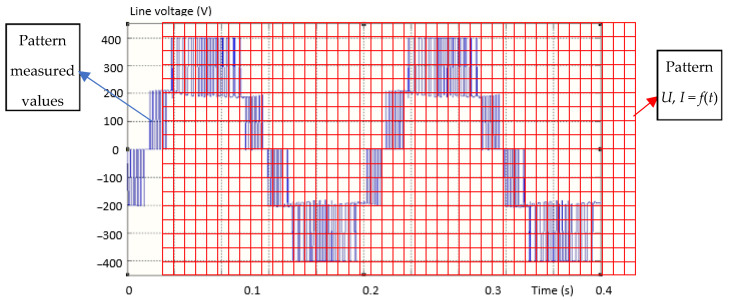
Explanatory graphic representation for the method of obtaining the specific differences necessary for the identification of the fault (I).

In Figure 7, the method applied to compare the measured values with the calculated ones is presented graphically.

**Figure 8:**
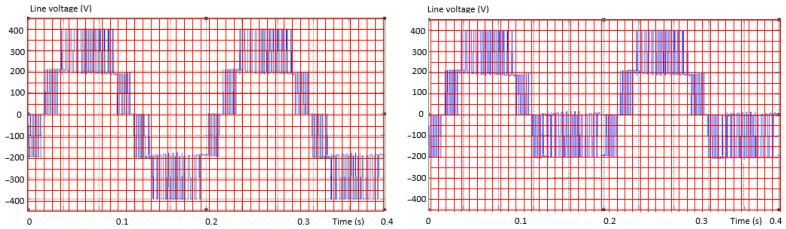
Explanatory graphic representations for the method of obtaining the specific differences necessary to identify the fault (II).

The measured values are compared with the “pattern” of the calculated predicted values. The procedure is graphically suggested in Figure 8.

All three techniques—the one based on the Park vectors, the one based on the value of the currents, and the one based on the spectral analysis—are used to detect and locate the faults of the power components, such as open circuits or short circuits in 3L inverters. It should be noted that the three techniques require only three current and three voltage sensors, presenting an advantage in terms of cost and implementation compared to other current techniques used.

A harmonic analysis is also performed to establish the existence of a fault in the analyzed 3L inverter. For external faults, the measured values of the currents and voltages are compared by hardware with the preset maximum values. Both actions cause the inverter control pulses to be blocked.

Faults in the power components of multilevel inverters change the shape of the voltage as well as the output current. Spectral analysis is performed for both output line voltage and current. THD analysis within the proposed method is used in real-time only for the quick identification of the fault condition of the inverter. In the off-line system, the spectral analysis allows the precise identification of the defective component by measuring and comparing the fundamental value and the harmonics determined for the signals obtained from the voltage and current transducers with the known and expected values. The existence of a d. c. component detected with the help of harmonic analysis is equivalent to the existence of a short-circuited power component. The values of each harmonic rank are compared within the method with the corresponding expected and known pattern. The differences between these values become significant in the case of each type of defective component.

The current spectral analysis has an additional advantage in terms of fast detection time (5 ms) compared to the other two (6 and 10 ms, respectively). A comparative analysis from the point of view of the reaction time is presented in Table 1. The times were obtained by direct measurement and are represented by the time from the appearance of the fault to its detection by the system. A disadvantage of this method is the non-specificity of fault identification.

Figure 7 and Figure 8 are the explanatory graphic representations of the proposed method. The pattern (the grid drawn in red) graphically represents the uncompleted, unwritten matrix, which will have either measured or calculated values in given time intervals. Measured or calculated (known) current or voltage values are filled in each grid box. Each value in the “box” will be compared, one from one matrix with the other from the other matrix (but the same one, at the same time). The differences will be interpreted by the method’s algorithm according to a preset error value. The superimposition of the grid drawn in red over the waveform (of the voltage but similarly for the current) from the output of the inverter suggests the principle of the method by assigning numerical values to each location, which actually represents the terms of the two types of matrices.

## 4. Simulation Results and Discussion

**Figure 9:**
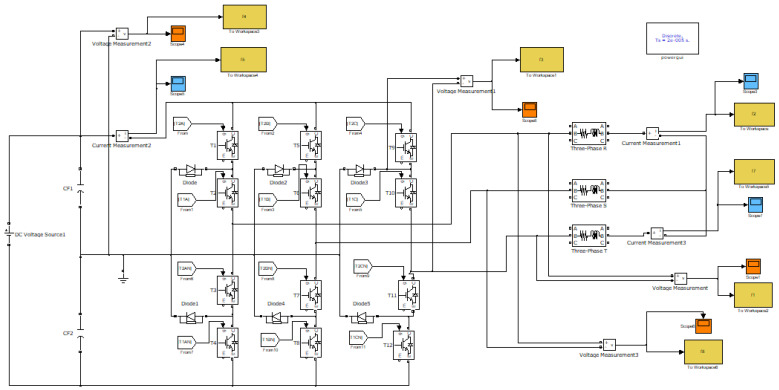
Schematic used to simulate fault conditions (Simulink).

The simulation of the failure mode operation of the power components—IGBTs, power diodes and capacitors—was carried out using the multilevel inverter with a 3L NPC structure scheme created in Simulink (The MathWorks). The main parameters of the scheme and the dynamic behavior of the system can be seen in Figure 9.

Transistors of (+) are marked according to the scheme (T1, T5, T9), (T2, T6, T10). Transistors of (–) are marked according to the scheme (T3, T7, T11), (T4, T8, T12). Thus: TRST1—(T1, T5, T9); TRST2—(T2, T6, T10); TRST3—(T3, T7, T11); TRST4—(T4, T8, T12).

The “1” state is associated with the on-state (saturated) transistor, and the “0” state is associated with the blocked transistor (off-state). The state values are presented in Table 2.

To perform the simulations, schematics were created with the specification of the particular type of fault. Thus, simulations were performed for situations in which a transistor was considered faulty and was replaced with an open circuit. For the situation in which it was considered short-circuited, then it was replaced with a continuous electric circuit. The defects of diodes and capacitors in the direct current intermediate circuit were also treated in the same way. To fully check all possible situations, faults were considered one by one for one or more components both on the plus and minus arms, for one or more phases.

**Figure 10:**
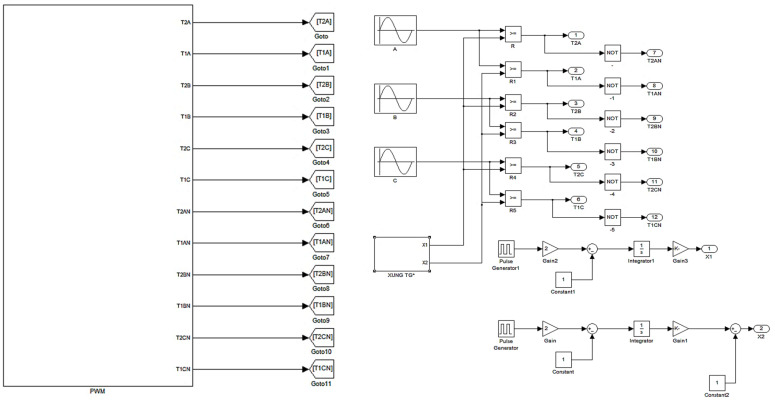
The Simulink module made to generate the PWM control pulses used in the 3L three-phase inverter.

Figure 10 shows the Simulink model made to generate the PWM control pulses used in the 3L three-phase inverter.

In a 3L three-phase inverter, there are 12 IGBTs, six balancing diodes and (can be considered) two capacitors. Under these conditions, considering that the faults (defects) that appear in a 3L inverter can manifest themselves in a single component, in two, in three, … in all 20, the number of possible situations is very large:

(31) $${N^{20}}_{DEF}=C_{20}^{1}+C_{20}^{2}+C_{20}^{3}+C_{20}^{4}+\ldots .$$

If up to two fault components are considered simultaneously, the number of situations that should be analyzed is:

(32) $${N^{2}}_{DEF}=C_{20}^{1}+C_{20}^{2}=20+190=210\;\mathrm{variants}$$

**Figure 11:**
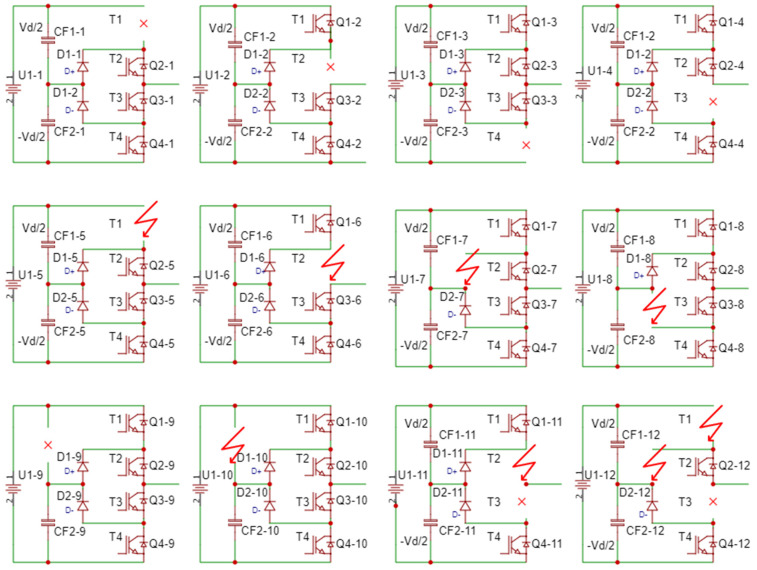
Basic internal fault situations through open-circuit fault and short circuit.

Figure 11 presents some of the basic internal fault situations through open-circuit fault and short circuit for one, two or at most three fault components per inverter arm (examples). The situation where there is at most one defective component was analyzed to exemplify the method. Additionally, all possible variants of the individual failure possibilities of the power components were considered to highlight the specific differences between the behaviors of the multilevel inverter in different failure situations.

The large number of possibilities and fault combinations limited the presentation of the method only to the simulation of the relevant ones. However, the method includes all defective situations in order to achieve the proposed goal.

**Figure 12:**
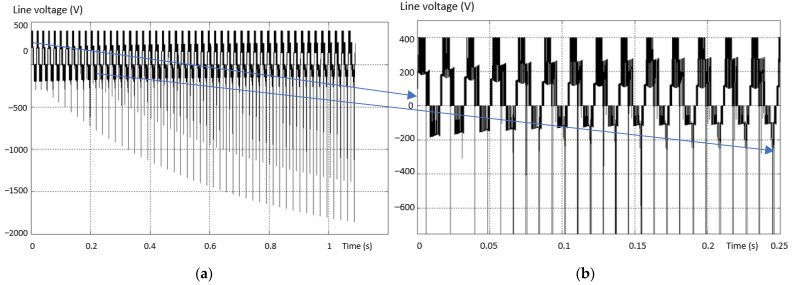
Voltage between two phases (line), valid phase—defective phase for the “+” lower transistor (**a**) and a zoom in the output voltage area (**b**).

The first analyzed situation has an open-circuit fault, simulated by the absence of the upper transistor on the “+” arm (first variant of the schematic in Figure 11). Figure 12 shows the voltage between two phases, one complete and valid and one fault. Figure 12b presents a zoom in the output voltage area in the same fault situation. There is an absence of a “tension level” compared to the normal situation. Supply is also through the diode plus the transistor to *N*.

**Figure 13:**
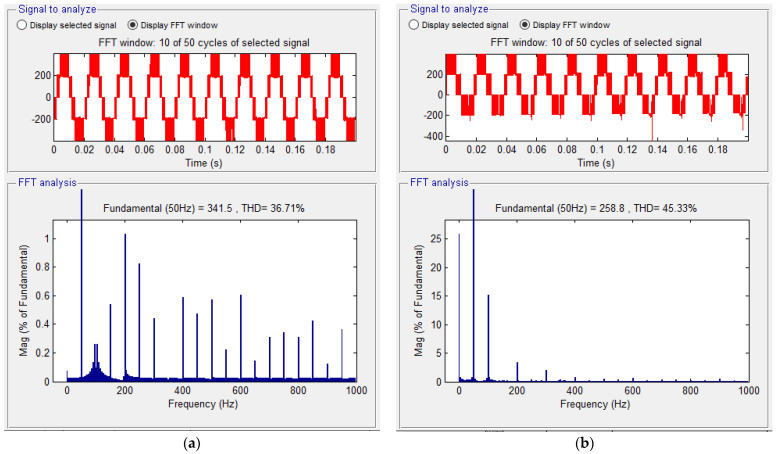
Comparative spectral analysis between (**a**) a voltage signal without a fault and (**b**) the fault situation.

Figure 13 shows the spectral analyses in the situation without defect (a) and the situation with defect (b). A lower THD and a higher fundamental component are found in the situation where the inverter is valid.

**Figure 14:**
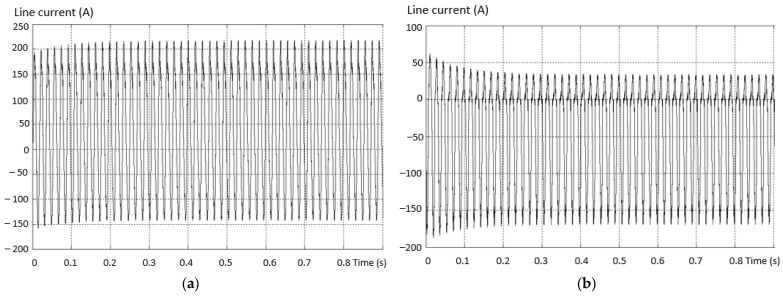
Variation of the phase current in the situation: (**a**) the current on the valid phase; (**b**) the current on the faulty phase.

Considering the same fault situation, a detectably different behavior is found for the inverter current that differs between the valid and the fault phase. The variation curves of the phase current in the situations (a) the current on the valid phase and (b) the current on the fault phase are presented in Figure 14.

The maximum value of the positive alternation corresponding to the fault inverter arm has a smaller amplitude than the negative one (which is complete for this type of fault). An asymmetry of the two shapes, both on the valid phase and on the phase where the fault exists (where it is more accentuated), was also found.

A comparative spectral analysis performed on the two phase currents when the inverter is valid and when there is an open-circuit fault shows differences in both amplitude and also THD. The amplitude of the fundamental measured in the case of the valid inverter has a higher value than that in the fault situation, and the THD in the fault situation is higher than that for the case when the inverter is perfectly functional.

**Figure 15:**
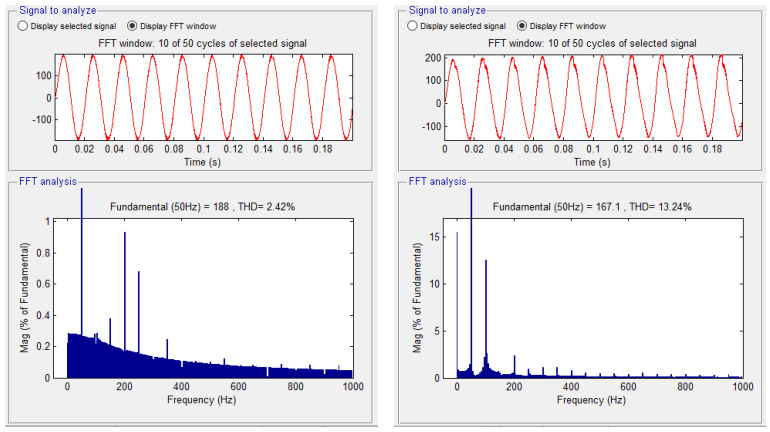
Comparative spectral analysis between a phase current on a valid phase and fault phase.

A comparative spectral analysis between a phase current on a valid phase and a faulty one is provided in Figure 15.

**Figure 16:**
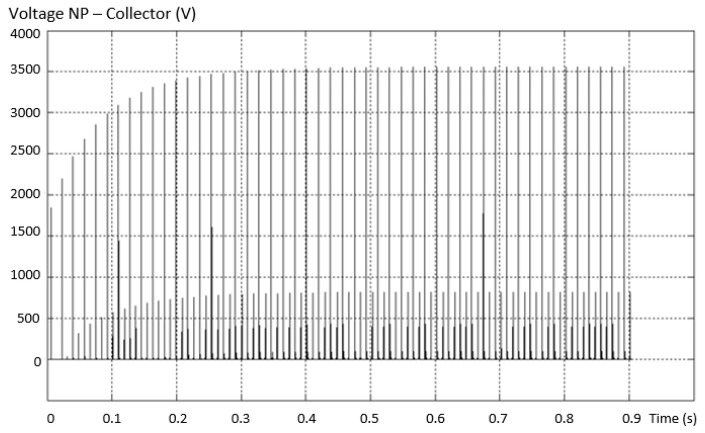
Evolution of the voltage between the neutral point and the collector of the valid transistor.

Considering the same fault situation, the evolution of the voltage between the neutral point and the collector of the valid transistor is shown in Figure 16. In this situation, the appearance of dangerous voltage peaks is observed.

**Figure 17:**
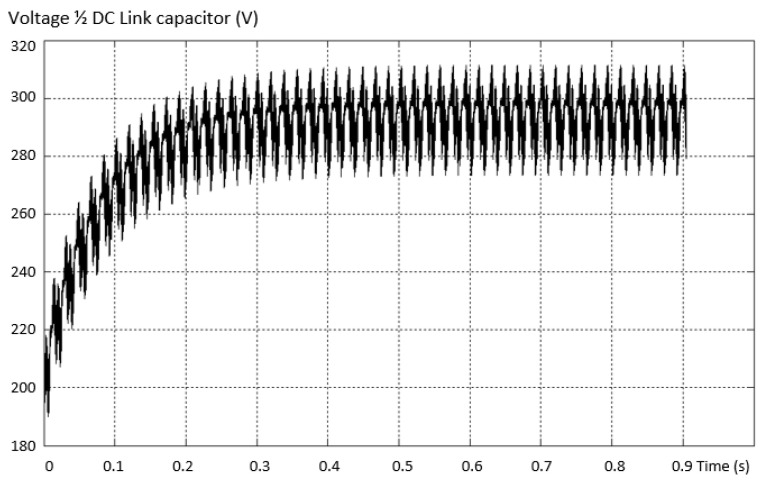
Voltage variation on a DC link capacitor.

In this type of fault, the voltage on one capacitor (one of the two connected in series) exceeds the value *V_dc_*/2, i.e., 200 V. Working in this failure mode endangers the integrity of the capacitors, unless they have an increased working voltage. The voltage variation across a DC link capacitor is shown in Figure 17.

**Figure 18:**
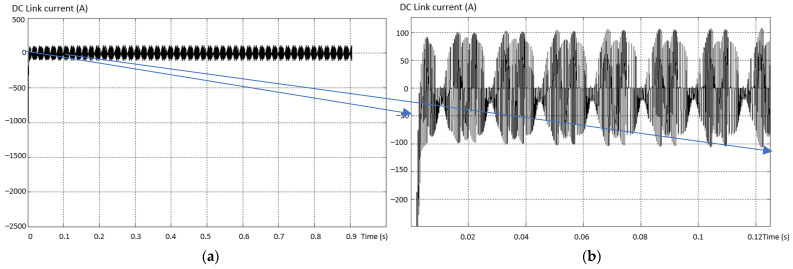
Current variation curves in the intermediate circuit (**a**) and zoom in the initial zone (**b**).

In the fault situation analyzed, the current in the intermediate circuit has no dangerous values and no important changes to be able to represent a criterion for determining the faults of multilevel inverters. The waveform variation curves of the current in the intermediate circuit in the fault situation considered by isolating the upper transistor in the “+” arm of the 3L inverter are presented in Figure 18.

**Figure 19:**
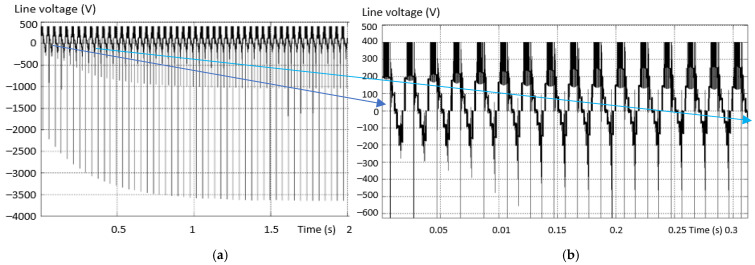
Line voltage representation between a valid and a faulty phase (**a**) and zoom (**b**).

The second fault situation was realized by considering it as a fault by isolating the lower transistor on the “+” bar of the multilevel inverter. The second fault situation determined a new set of diagrams that validated the conclusions obtained after the first simulations. Figure 19a presents the voltage between two phases, one complete and valid and the other with fault. The fault situation presented there is an open-circuit fault, simulated by interruption, by the absence of a transistor on the lower “+” arm (the second scheme from Figure 11). Figure 19b shows a zoom in the output voltage area in the same fault situation. There is a lack of a “voltage level” compared to the normal situation.

Considering the fault situation under discussion, the difference between the valid phase and a fault phase can be determined and is similar in value to the first analyzed situation.

**Figure 20:**
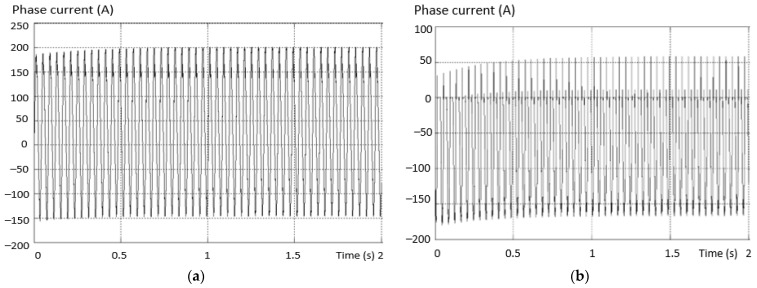
Current waveforms on two phases of an inverter with the defect from the 2nd situation. (**a**) The current on the valid phase; (**b**) the current on the fault phase.

The waveform variation of the phase current in situations (a) the current on the valid phase and (b) the current on the fault phase are presented in Figure 20.

The maximum value of the positive alternation of the current corresponding to the defective inverter arm has a smaller amplitude (practically zero) on the phase containing the fault transistor. It was also found that there is an asymmetry of the two alternations both on the valid phase and on the phase where the fault exists, and it is more accentuated. Moreover, on the valid phase, the positive alternation has a greater amplitude than the negative one.

**Figure 21:**
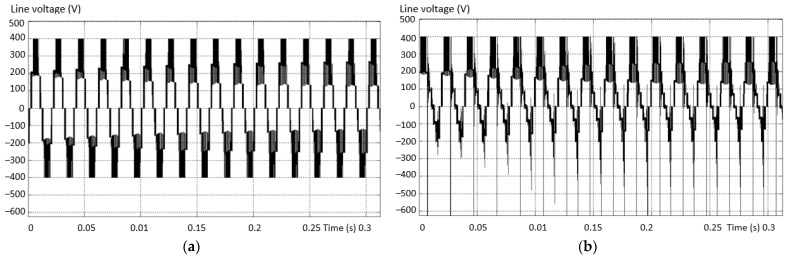
The waveforms of the line voltage in the non-fault situation (**a**) and the fault situation (**b**).

Figure 21 shows the waveforms of the line voltage in (a) the fault-free and (b) the fault situation. There is a lack of a “voltage level” compared to the normal situation when the inverter is valid. The shape of the voltage is similar to that of the first case analyzed.

**Figure 22:**
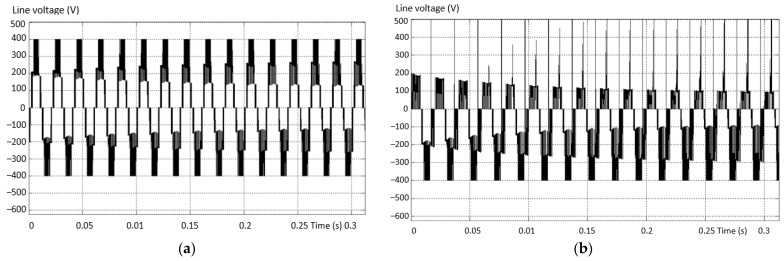
Voltage between a valid phase (**a**) and a defective one (**b**) (transistor interrupted on the minus side).

In order to be able to validate the investigation method, another defect scenario was considered: “−” transistor is missing, similarly with the third variant from Figure 11. Figure 22 shows the evolution of the line voltages (a) between two valid phases and (b) between one phase non-fault and one with fault (on the minus side). From this analysis, it can be seen that the levels of the two voltages are different. As in the first scenario, when a transistor is interrupted, a voltage level is missing from the alternation opposite to the missing transistor.

**Figure 23:**
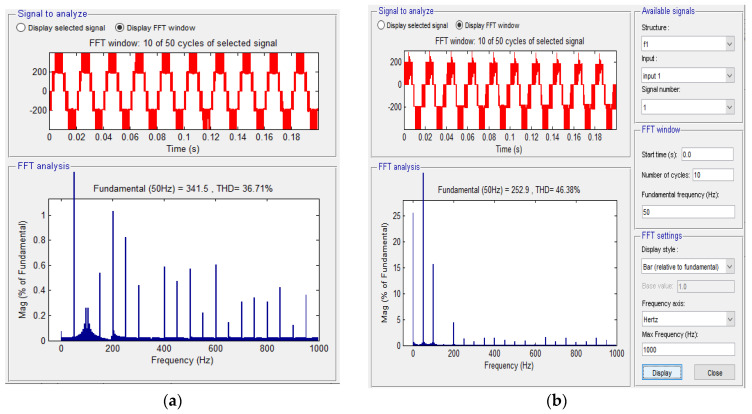
Comparative analysis: (**a**) a voltage signal without fault and (**b**) the fault situation.

Figure 23 shows the spectral analyses in the case (a) without defect, and in the case (b) with fault. A lower THD and a higher fundamental component are found in the situation where the inverter is valid. The increase in the THD, respectively the decrease in the fundamental amplitude, in the case of a fault, is also a fault indication in this scenario.

**Figure 24:**
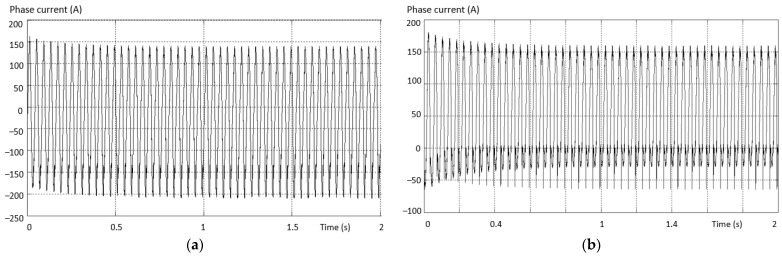
The waveforms of the current on two phases of an inverter with the defect from the 3rd situation: (**a**) the current on the valid phase; (**b**) the current on the faulty phase.

Figure 24 illustrates the waveforms of the current on two phases of an inverter with the defect in the third situation: (a) the current on the valid phase, and (b) the current on the fault phase. The maximum amplitude of the phase current for the positive alternation corresponding to the defective inverter arm (containing the defective transistor) has a larger amplitude than the other phases. It is also found that there is an asymmetry of the two alternations both on the valid phase and on the fault phase. Moreover, on the valid phase, the positive alternation has a smaller amplitude than the negative one.

**Figure 25:**
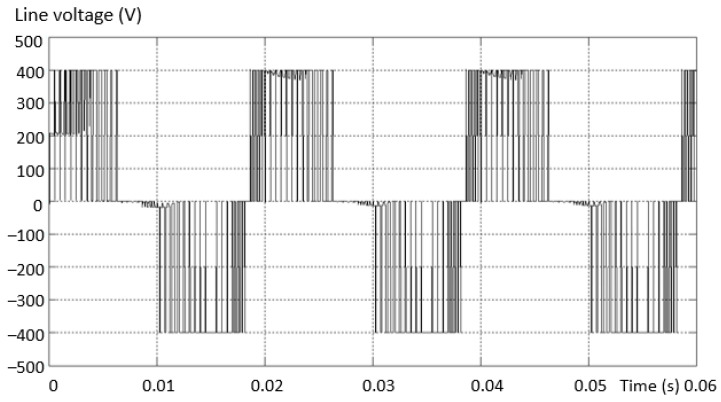
Voltage between a valid phase and a faulty one with internal short circuit.

In the fourth fault situation analyzed, the short-circuit fault of a “+” transistor was considered, as in the fifth variant in Figure 11 (the first diagram on the second row). Figure 25 shows the line voltage between a valid and a fault phase with an internal short circuit installed (top transistor short-circuited). There is a lack of a level voltage on both the positive and negative alternation. In this way, the entire supply voltage is applied to the remaining valid transistors, endangering their integrity. In general, the transistors in the multilevel inverters are not sized in terms of voltage at the full supply voltage (usually they are sized at *V_dc_*/2).

**Figure 26:**
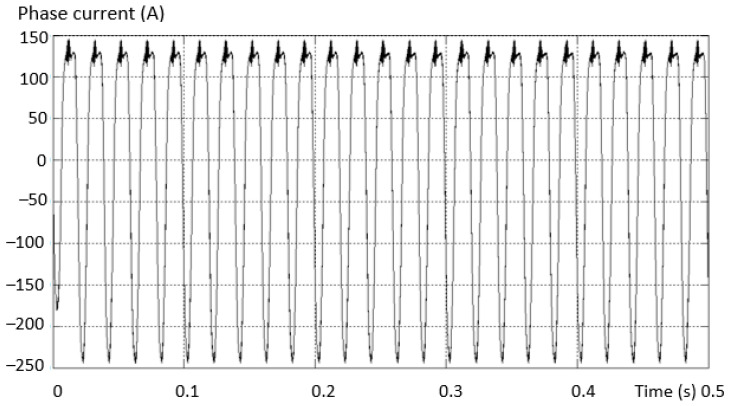
Current on the faulty phase.

In the case of an internal short circuit, the imbalance between the alternating phase currents is smaller. The positive alternation is more distorted and has a smaller amplitude than the negative one. Phase current is shown in Figure 26.

**Figure 27:**
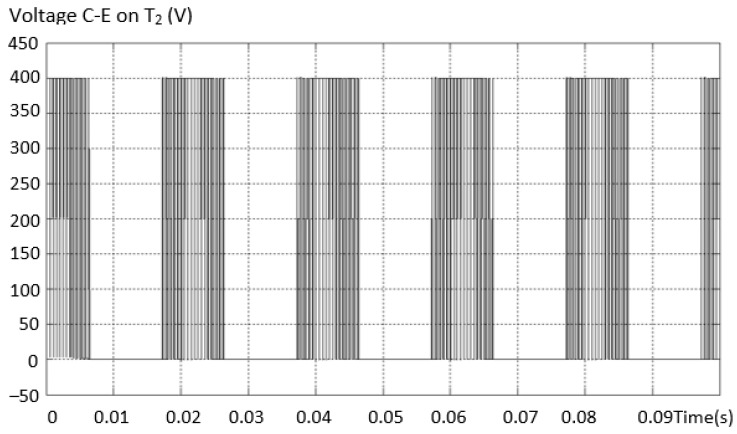
The voltage on T_2_ in the circuit of the arm where the short circuit is installed.

To check the dimensioning of the power transistor (IGBT), the voltage on T2 was also determined in the arm circuit where the short circuit was installed, and it was found that it takes all the supply voltage *V_dc_*, as in Figure 27.

**Figure 28:**
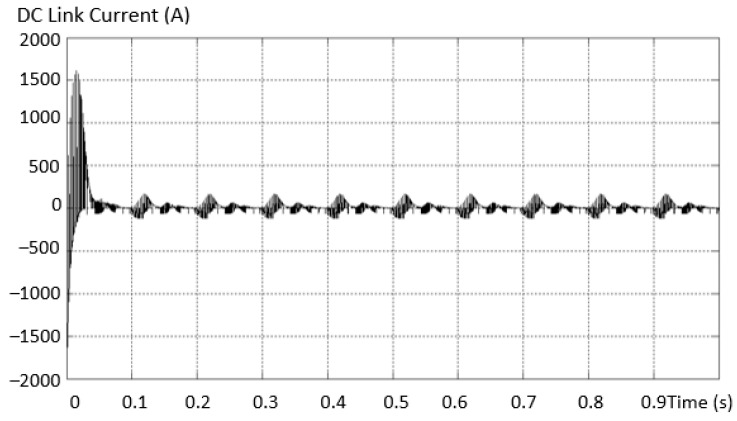
Current evolution in the DC intermediate circuit for a short-circuited transistor.

Further, for dimensioning and checking the parameters, the current in the DC intermediate circuit for the short-circuited transistor was also determined, as in Figure 28. A very high current value of ~ 1500A is found at the initial moment, and practically the capacitor corresponding to the short-circuited transistor is discharged and fully charged with each PWM pulse.

**Figure 29:**
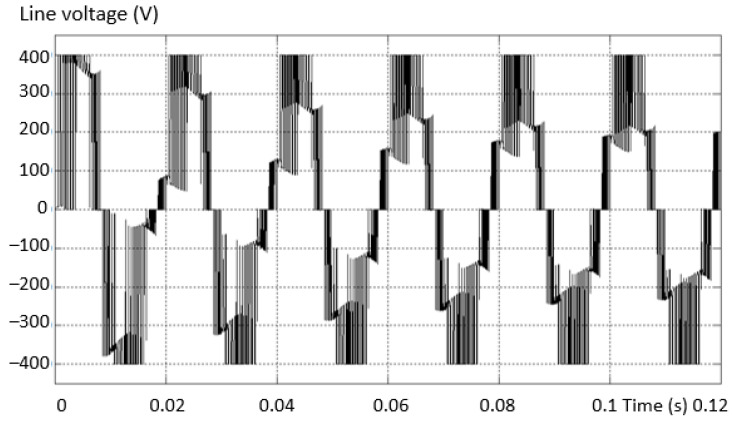
Line voltage between a valid phase and a faulty one with an interrupted capacitor.

In the fifth fault situation analyzed, the fault in the DC link circuit was considered. It was considered in the first stage that the capacitor on the “+” line is broken, as in the ninth variant in Figure 11 (the first diagram on the third row). Figure 29 shows the line voltage between a valid phase and a faulty one with the capacitor disconnected toward “+”.

**Figure 30:**
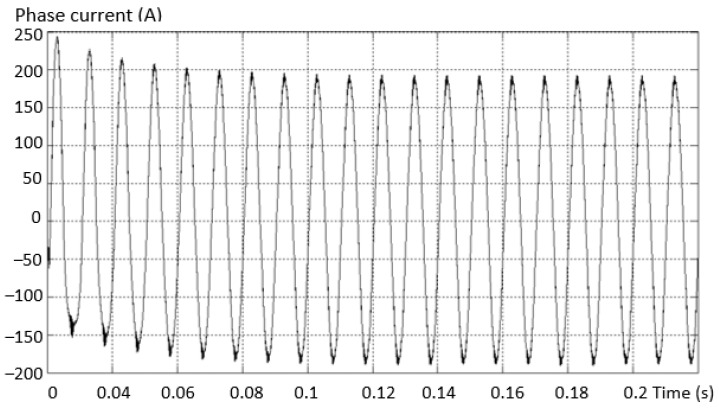
Output current shape per phase.

The existence of the three voltage levels is found, but an increase in the THD of the output voltage is determined. The variation of the output current per phase is shown in Figure 30. It is found that there is a damped process with higher overshoot values toward the “+” terminal (on the missing capacitor).

**Figure 31:**
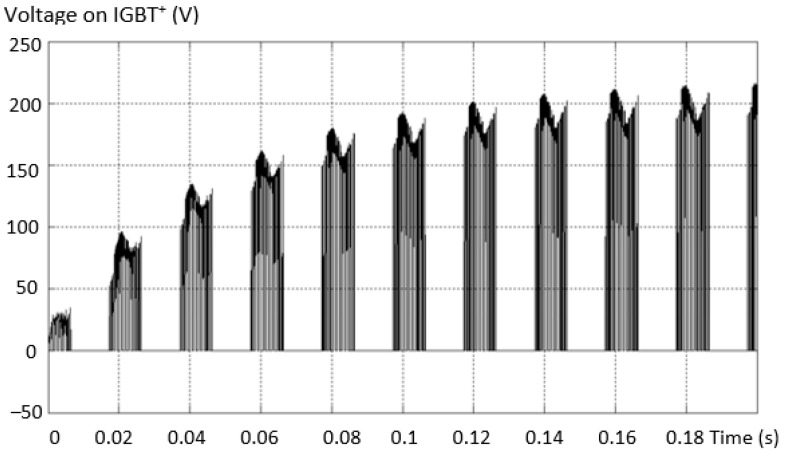
Waveform of the voltage on the transistor from the “+” terminal in case of a broken capacitor type fault.

**Figure 32:**
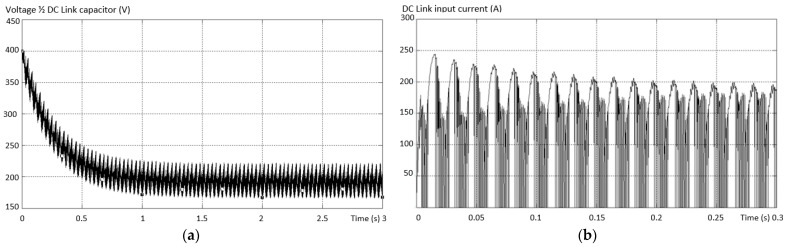
The waveforms (**a**) of the voltage in the DC link circuit at the terminals of the missing capacitor circuit; (**b**) the variation of the input current in the inverter.

For testing and dimensioning, the voltage on the transistor from the “+” terminal was also monitored. The voltage variation is shown in Figure 31. The amortized process determined by the variation of each parameter, voltage or current in case of interruption of the capacitor from the “+” terminal can also be seen in Figure 32.

Variation of the voltage in the DC link circuit at the terminals of the missing capacitor circuit is shown in Figure 32a where it decreases from the maximum value of the Li-Ion battery up to half of it, which also represents the normal operating value. If the other parameters do not immediately endanger the integrity of the inverter, causing only the degradation of the signals provided at the output, the input current in the inverter has a dangerous defect evolution. The value of the dc input current can generate, over time, defects with the interruption of the inverter operation due to the thermal effect, both the maximum value and the ripple, as in Figure 32b.

**Figure 33:**
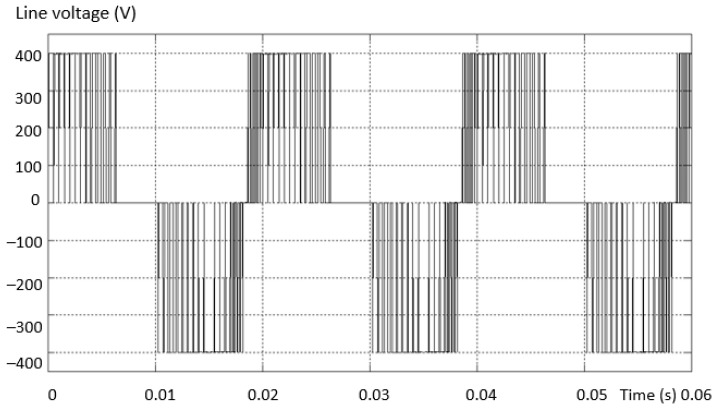
Line voltage, between a valid and a faulty phase with short-circuited capacitor.

**Figure 34:**
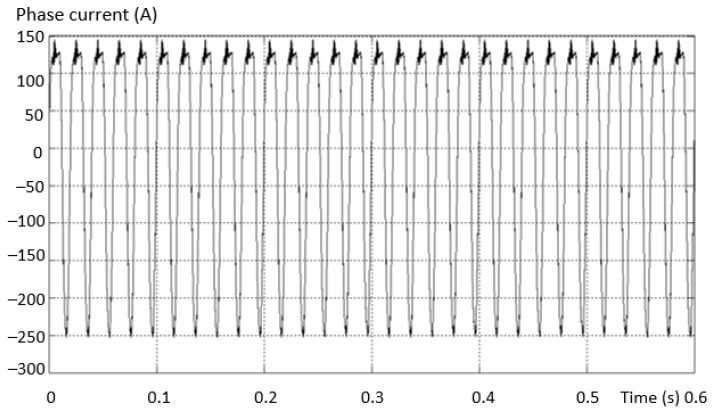
Output current in the fault situation—short-circuited capacitor.

In the sixth fault situation analyzed, another type of fault in the DC circuit was considered. It was taken into account that the capacitor is short-circuited to the “+” line, as in the 10th version in Figure 11 (second diagram on the third row). Figure 33 shows the line voltage between a valid phase and a faulty one with a short-circuited capacitor (toward “+”). There is a lack of voltage levels at the output, and the line voltage is the same as with the 2L inverters. The output phase current is unbalanced on the positive alternation and the amplitude is lower on the side of the short-circuited capacitor. The variation of this current is shown in Figure 34.

**Figure 35:**
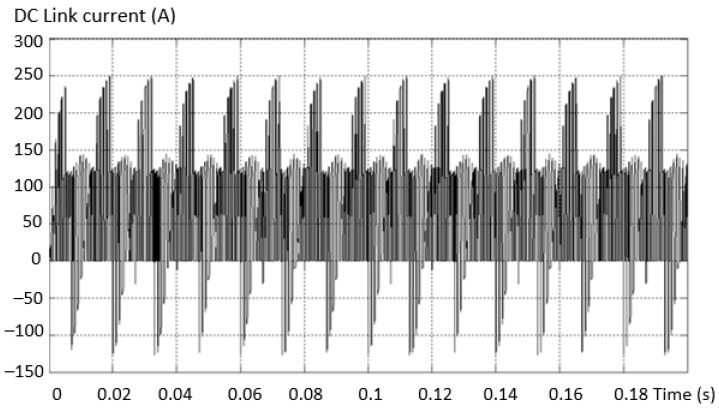
The current in the intermediate DC link circuit at the capacitor short-circuit fault.

The current in the intermediate circuit DC link to the short-circuit defect of the capacitor from the “+” terminal shows a large ripple and a positive and negative variation of the values, as in Figure 35. This behavior of the current determined by this type of defect is very dangerous due to the destructive potential of recurrent faults.

**Figure 36:**
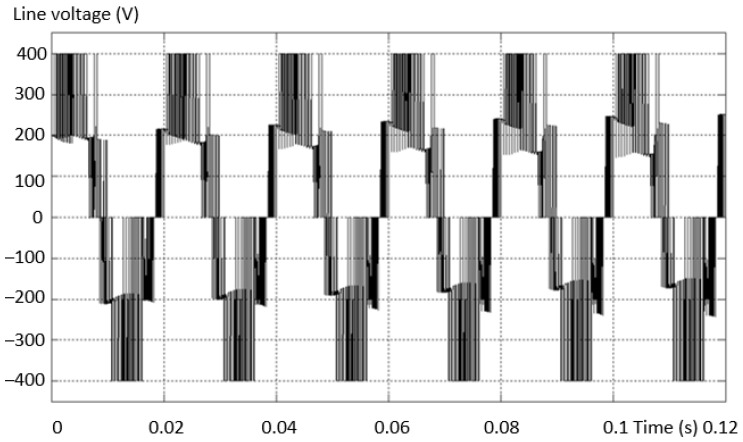
Line voltage between a valid and a faulty phase with an interrupted diode.

In the seventh fault situation analyzed, another type of fault was considered by interrupting the balancing diode in the “+” arm. Figure 36 shows the line voltage between a valid phase and a faulty one with interrupted balancing diodes (toward “+”). The presence of voltage levels at the output is found, but it is different from the correct pattern, which causes the increase in THD.

**Figure 37:**
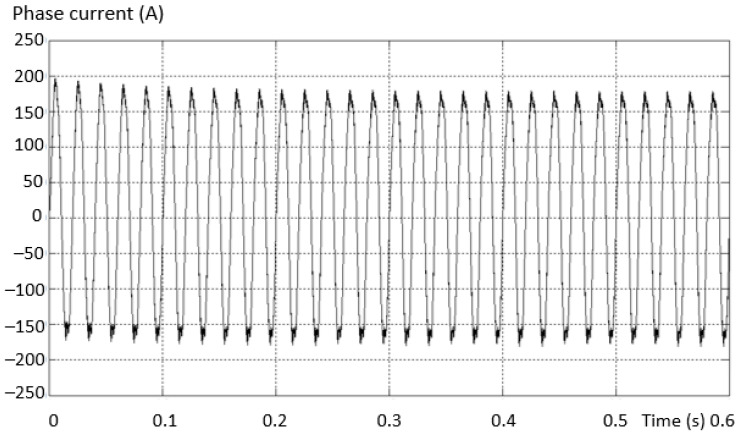
Output current in the fault situation—broken diode (open circuit).

The output phase current is balanced, but with a higher THD than in inverters without fault. The variation of this current is shown in Figure 37.

**Figure 38:**
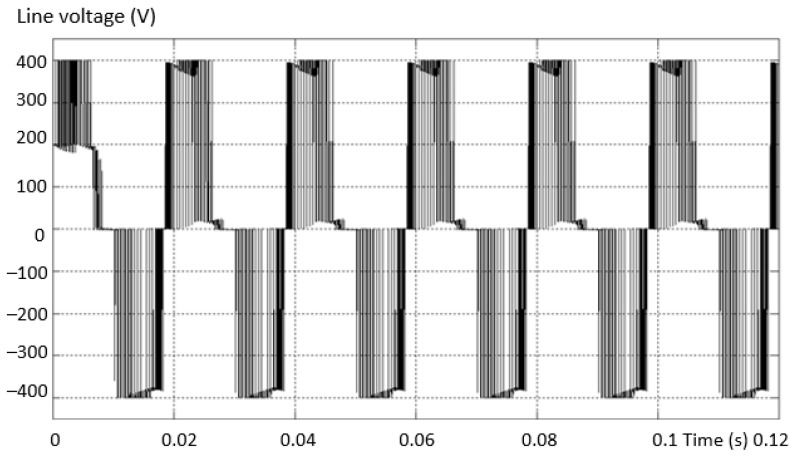
Line voltage between a valid and a fault phase with short-circuited diode.

**Figure 39:**
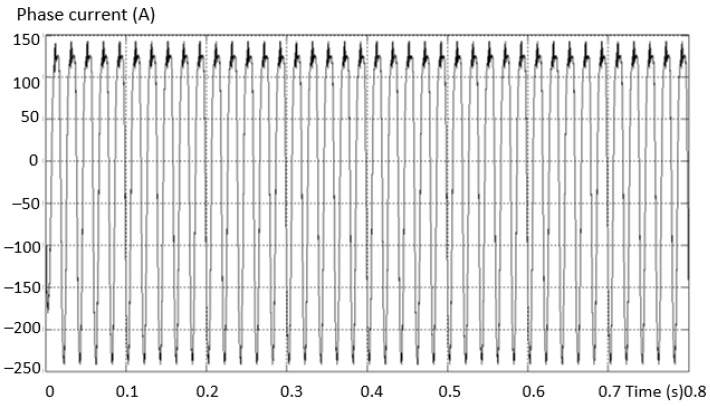
Phase output current with short-circuited diode.

In the eighth situation analyzed, a fault was considered due to the short-circuiting of the balancing diode in the “+” arm, as position 7 in Figure 11 (the third in row 2). Figure 38 shows the line voltage between a valid phase and a fault one with short-circuited balancing diodes (inverter arm toward “+”). The three voltage levels are missing, so the output voltage is similar to that of the 2L inverters. The output, phase current, is unbalanced, with a higher THD than in fault-free inverters. The variation of this current is shown in Figure 39. The amplitude of the negative semi-alternation is greater than that of the positive one (the short circuit is installed on the positive arm of the inverter).

## 5. Experimental Verification

**Figure 40:**
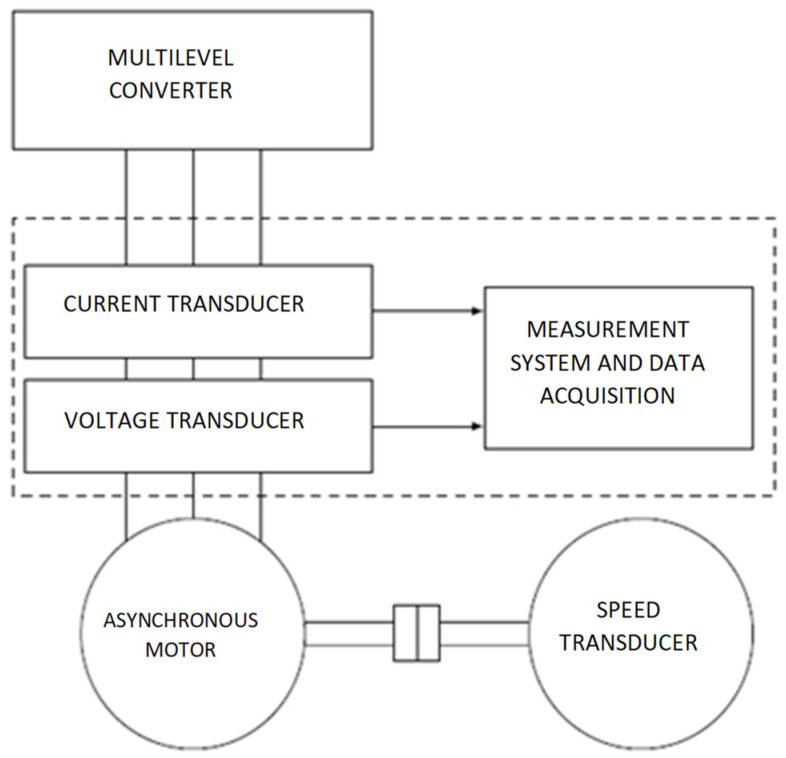
Test bench block diagram.

For the implementation and verification of the proposed fault detection method, a test stand was used as in Figure 40, where the test bench block diagram is provided. This test bench was initially conceived in the project TISIPRO POC-A1-A1.2.3-G-2015, ID_40_416, no. 61/05/09/2016, 2016–2021—“Software solutions for controlling multilevel converters with high energy efficiency”, and it was further developed in the form presented in Figure 40, in order to test several failure scenarios.

The cells were organized in battery packs (Nissan Leaf type 2nd generation 24 kWh) and used to power the system. The maximum voltage value is found in most current EV powertrains.

Battery specifications
Rated voltage7.40V;Minimum voltage6.00V;Maximum voltage8.30 V;Battery capacity60 A.

Features of the battery module
Dimensions300 mm × 222 mm × 34 mm;Weight3.65 kg;Number of modules48.

The tested motor was an asynchronous type used when equipping an EV and had the following key features:

300 Nm peak torque;
125 kW peak, 45 kW continuous motor power;
125 kW peak, 41 kW continuous generator power;
Full power at 300–430 VDC;
EV/HEV traction drive or HEV starter/generator system.

The measuring system was based on Hall type voltage and current transducers with good response speed and accuracy.

The sensor system for measuring the output parameters was made up of three voltage sensors and three Hall current sensors. The instantaneous values of line currents and voltages at the output of the multilevel inverter were measured with these sensors. Measuring the parameters (voltage on the dividing capacitors and the total input current) of the intermediate circuit still required a current transducer and two voltage transducers. The use of these three additional sensors was justified by the substantial improvement in the performance of the method for identifying the faulty power component.

These sensors had the following general performances:

- overall accuracy < +0.5%
- linearity error < 0.2%
- response time < 1 μs

Depending on the voltage level and the power of the monitored inverter, sensors with appropriate parameters were used.

**Figure 41:**
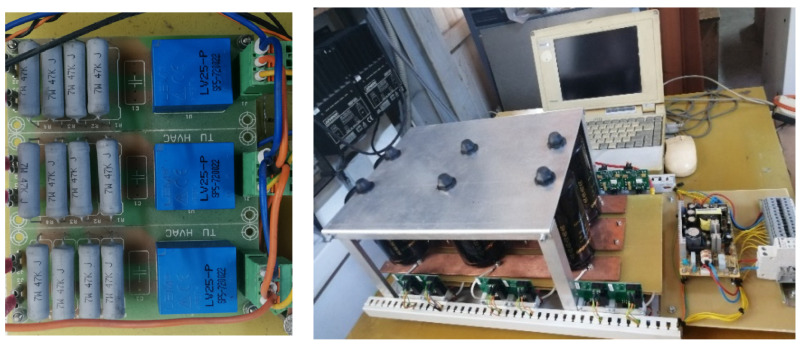
Measurement system for three-phase system. View with the 3L inverter.

**Figure 42:**
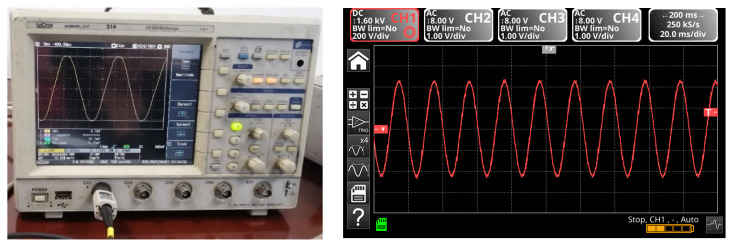
Current signals at the output of the three-phase multilevel inverter.

The multilevel three-phase inverter prototype (3L) was made with IGBT transistors with low losses, and the intermediate circuit was made of 6 pairs of electrolytic capacitors mounted as in Figure 41. The capacitors in the intermediate direct current circuit were connected three each in parallel and formed two groups in series just like in the simulated system. The load connected to the output 3L of the three-phase inverter was a motor, and the switching frequency was chosen at the value of 4 kHz; the waveform of the current was with less distortion, being close to sinusoidal as in Figure 42.

The practical solution is presented as a basic element that uses configurable subsystems to switch between architectures.

By using the fault detection strategy, it is possible to avoid any misdiagnosis that could result from the existence of electrical switching noise, sensor response time, power component switching delay time and measurement errors. The presented method does not require additional sensors compared to the normal control circuits of an inverter.

The waveforms obtained in the normal operating state of the inverter compared with the waveforms specific to the failure states were determined with results similar to those illustrated in the simulations. A further research goal is to create an independent functional device based on the presented method that can be simply connected and protect any multilevel inverter without interfering with its control.

To realize the effective real-time protection of the inverter and for a universal use of the system, the transfer of direct electrical signals was preferred in favor of some software program sequences when a defect is detected. These signals ensured the quick start/stop condition of the protected inverter.

**Figure 43:**
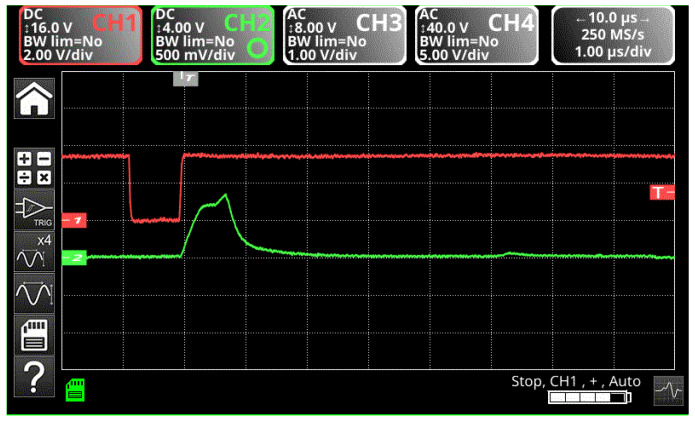
Impulse blocking when an internal fault is detected.

The signals were galvanically separated in order to not cause disturbances in the operation of the inverters that provided protection. The detection of a fault situation causes the production of a pulse (channel 1), which causes the immediate blocking of the first control pulse on the IGBT gate (channel 2) in less than 1μs to be able to optimally protect the inverter (as in Figure 43). The signals are transmitted between the control section of the fault detection system and the power section of the inverter, using a system with galvanic isolation.

Compared to the situations presented when the existing voltage and current transducers were used in the system, a special situation was the monitoring of defects in the direct current intermediate circuit. In this situation, it was necessary to mount two voltage Hall transducers in parallel with the capacitors in the power circuit.

**Figure 44:**
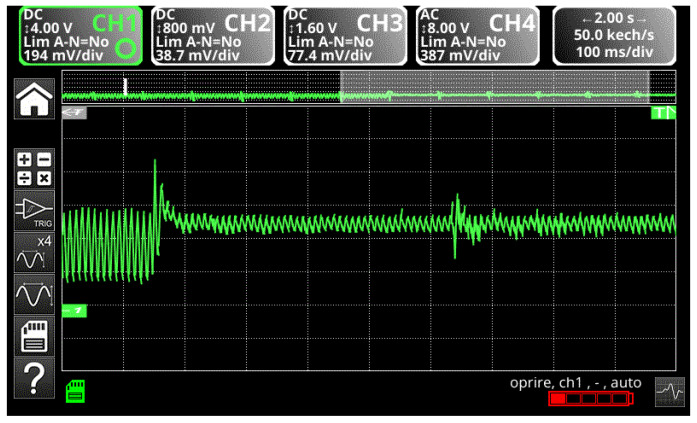
The waveform of the voltage in DC link circuit at the terminals of the missing capacitor.

The behavior of the voltage on ½ of the capacitor bank in the dc link is checked when a capacitor in the intermediate circuit suddenly breaks during operation. The voltage behavior is similar to that obtained by simulation and is presented in Figure 44.

**Figure 45:**
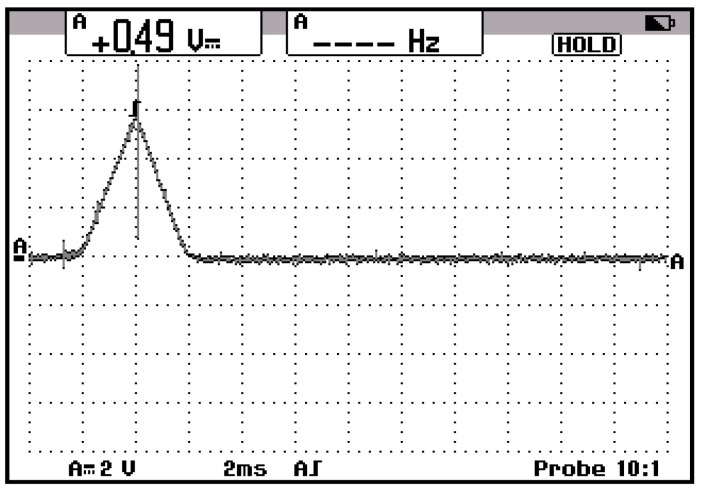
The waveform of the short-circuit current between two phases of the multilevel inverter.

To protect against external faults, a short circuit was made between two output phases, and the total reaction time was determined by measuring the phase current. The current variation is shown in Figure 45. The recording in Figure 45 has a time base of 1μs/div. A very fast reactive speed of protection was achieved with the system based on the method of identifying defects in multilevel inverters.

Open-circuit faults were realized by removing the component considered defective, and short-circuit faults were realized by short-circuiting the component. Taking into consideration that the experimental platform is under development, the completion of all defective situations is in progress.

## 6. Conclusions

For the maintenance of EV propulsion systems, it is necessary to accurately determine the fault components that may appear during the operation of a multilevel inverter. The method proposed in this work is characterized by simplicity and good adaptability to all topologies of multilevel inverters. The higher the number of inverter levels, the more difficult it becomes to identify the fault component(s). Under certain conditions, if an incipient fault situation is not identified in the shortest possible time, it is possible that the fault will continue to expand, causing a more serious fault situation that entails additional costs and time to restore the inverter to operation. Signals already available in the control system, such as load current and output voltage, are suitable for use in fault diagnosis. Simulations and experiments were performed to calibrate and verify the performance and effectiveness of the presented strategy. The values measured by the sensors can be transmitted remotely and analyzed in real time both when the vehicle is in motion and when the batteries are charged.

This innovative method allows the accurate detection of both internal defective components and external faults by using the same system of current and voltage transducers specific to any inverter, which is completed with the platform containing the voltage and current values matrix.

The proposed method and system allow complete and quick protection for both external and internal defects. The identification of the phase on which a short circuit has occurred or the lack of a phase of the motor is found by comparing the value measured by the sensory system with the expected and known value from the matrix of correct values. The comparison is performed in hardware by using voltage comparators included in the system. The system ensures the detection of all external defects, such as a short circuit between the phases of the asynchronous motor or an accidental connection between a phase of the motor and the plus or minus terminals of the EV’s power battery.

The most dangerous of the external faults of the inverters are the short circuits because the values of the defect parameters evolve to very high values in a short time. In the event of an external short circuit, the maximum current protections must act in a time < 10 μs in order to prevent failures of the IGBTs. The proposed system also includes hardware comparators that act on the protection system in a time determined by the type and quality of the transducers; generally, this is <5 μs.

A specific feature of the method is the detection of faults in the DC intermediate circuit by discovering the interruption, respectively the short-circuiting of the capacitors and the defects in the balancing circuit, and respectively the power diodes.

The protection evaluation results in digital format allow the system to be connected to IoT devices that transmit the obtained results to automated devices that also perform troubleshooting of the power modules. In this way, the entire process of fault identification and troubleshooting is automated. The collected data can be organized in databases and used later to predict the occurrence of defects.

The proposed method has a role in preventive maintenance and in the safe operation of the EV. If an evolving electric defect of the traction inverter can be identified in a short time, the safety of the passengers is not put at risk, for example, by a defect in the situation of overtaking or entering an intersection. An important application of the method will certainly be the autonomous operation of the EV when an automatic system will have to make decisions regarding the safety of the passengers.

Regarding future developments to improve the system functionality and performance, several directions can be identified:

- completing the experimental verification of the method for all possible fault situations;
- expanding and completing the parametric matrices for all types of topologies of 3L inverters type ANPC, T-type NPC and hybrids;
- developing data acquisition systems with remote communication and action features, including the implementation of industrial IoT systems;
- using machine learning algorithms in future applications;
- designing sensor systems and real-time analog digital acquisition and conversion hardware using complex programmable logic devices (CPLDs) or field programmable gate arrays (FPGAs) depending on the application or the cost of the system.

## Figures and Tables

**Table 1:** A comparative analysis from the point of view of the reaction time.

Techniques	Hardware	Detection Time (ms)	Detection/ Localization	Quality and Performance
Stator current spectrum analysis	Three current sensors	5	Yes/ Poor	Very good
Average value of stator currents	Three current sensors	6	Yes/ Good	Good
Park stator current vectors	Three current sensors	10	Yes/ Good	Good

**Table 2:** State values for the multilevel inverter.

No.	Switch State				Output Phase Voltage	Remarks
	T_RST1_	T_RST2_	T_RST3_	T_RST4_	*U_outN_*	
1	“1”	“1”	“0”	“0”	*V_d_*	Positive (*Pos*)
2	“0”	“1”	“1”	“0”	0	Zero (0)
3	“0”	“0”	“1”	“1”	−*V_d_*	Negative (*Neg*)

## Data Availability

Data from simulations are available upon request.
